# HALP Score in Predicting Response to Treatment in Patients with Early-Stage Gastric Cancer: A Multi-Centred Retrospective Cohort Study

**DOI:** 10.3390/medicina60122087

**Published:** 2024-12-20

**Authors:** Tolga Köşeci, Mustafa Seyyar, Yasemin Aydınalp Camadan, Halil Çelik, Burak Mete, Hakan Demirhindi, Kadir Eser, Serdar Ata, Ali Alper Solmaz, Timuçin Çil

**Affiliations:** 1 Medical Oncology Department, Faculty of Medicine, Çukurova University, Adana 01330, Türkiye; yaydinalp@cu.edu.tr; 2 Medical Oncology Department, Gaziantep City Hospital, Gaziantep 27470, Türkiye; mustafaseyyar@saglik.gov.tr; 3 Medical Oncology Department, Faculty of Medicine, Mersin University, Mersin 33340, Türkiye; halilcelik@mersin.edu.tr (H.Ç.); kadireser@mersin.edu.tr (K.E.); 4 Public Health Department, Faculty of Medicine, Çukurova University, Adana 01330, Türkiye; bmete@cu.edu.tr (B.M.); demirhindi@cu.edu.tr (H.D.); 5 Medical Oncology Department, Afyon State Hospital, Afyon 03030, Türkiye; serdar.ata@saglik.gov.tr; 6 Medical Oncology Department, Adana City Hospital, Adana 01370, Türkiye; alialpersolmaz@saglik.gov.tr (A.A.S.); timucin.cil@sbu.edu.tr (T.Ç.)

**Keywords:** pathological response, HALP score, perioperative treatment, gastric cancer

## Abstract

*Background and Objectives*: The HALP (Haemoglobin, Albumin, Lymphocyte and Platelet) score is used to predict the prognosis of different types of cancer. This study aimed to investigate the role of the HALP score in predicting pathological response in early-stage gastric cancer patients. *Materials and Methods*: This retrospective cohort study was conducted on 118 patients diagnosed with early-stage gastric cancer and subjected to perioperative (FLOT) treatment between 2018 and 2023. The role of the HALP score in predicting the pathological response to perioperative treatment in patients was investigated. *Results*: The mean age of the 118 patients included in the study was 61.3 ± 11.1 (min = 23; max = 86). In the ROC analysis, the optimum cut-off value for the HALP score in pathological response classification was found to be 28.9 (AUC = 0.710, sensitivity = 56.7%, specificity = 80%, PPV = 86.79%, NPV = 46.15%). The pathological response rate was 69% in all patients, 87% in patients with a HALP score ≥ 28.9, and 52% in patients with a HALP score < 28.9 (*p* < 0.001). The probability of pathological response is 6.5 times higher in patients with a HALP score ≥ 28.9. In the Fagan nomogram, when the HALP score was ≥28.9, our pathological response probability estimate (post-test response probability) was found to increase to 64.8% (Positive Likelihood Ratio = 3, Negative Likelihood Ratio = 0.53). In patients with HALP scores ≥ 28.9 and <28.9, progression rates were 16.7% and 47.8%, respectively (*p* < 0.001), and median survival times were 45.4 and 30.6 months (*p* < 0.001). *Conclusions*: The HALP score is a useful and easily accessible score for determining pathological responses in patients with locally advanced gastric cancer.

## 1. Introduction

Gastric cancer ranks fifth among all cancers and third among cancer-related deaths. It is more common in men [1]. Although the incidence of gastric cancer is decreasing in Western countries, it remains a serious health problem [2]. The most common histopathological form of gastric cancer is adenocarcinoma. According to the Lauren classification, the two histological subtypes of gastric cancer are intestinal and diffuse types, which exhibit different clinical and molecular features [3,4]. Approximately 65–70% of gastric cancer is at the “locally advanced” or “advanced” stage at the time of diagnosis, indicating a poor prognosis [5]. The main treatment for early-stage disease is surgery. Adjuvant, neoadjuvant and radiotherapy treatment options can also be applied to these patients. Especially in patients diagnosed with gastric adenocarcinoma, the FLOT4 AIO study showed that survival and response rates were better in patients with locally advanced cancer if they received a perioperative (FLOT) regimen in the perioperative period when compared to other treatment options. Hence, the perioperative regimen was defined as a standard treatment option in the perioperative approach to gastric cancer treatment [6]. Some studies have shown that proliferation, invasion and metastasis have a relationship with systemic inflammation in cancer patients [7]. The resulting inflammatory response contributes to tumour growth [8]. During inflammation, some blood cells participate in the adaptive immune response by causing cytokine release, which contributes to tumour proliferation and growth [9]. It is known that inflammatory markers such as lymphocytes, neutrophils and albumin have prognostic importance in cancer patients [10]. Additionally, many inflammatory indices such as platelet–lymphocyte ratio, neutrophil–lymphocyte ratio, and prognostic nutritional index are used as prognostic markers in patients with gastric cancer [11,12]. The combination of multiple biomarkers allows for a more comprehensive assessment and potentially a more accurate prediction of disease outcomes. One of these combinations, the HALP score, is calculated using haemoglobin, albumin, lymphocyte and platelet values. The HALP score evaluates both the immune system and nutritional status, and has been shown to have prognostic significance in different types of cancer [13,14] and patients with metastatic gastric cancer [15].

However, no study is known to have evaluated the importance of the HALP score in patients receiving perioperative treatment. Our study aimed to investigate the HALP score in predicting pathological response and prognosis in patients diagnosed with gastric adenocarcinoma and treated with a perioperative regimen.

## 2. Materials and Methods

### 2.1. Research Type and Ethics

This retrospective cohort study included patients with locally advanced gastric cancer who applied to the Oncology Department of Çukurova University, Mersin University, Gaziantep City Hospital and Adana City Hospital and received perioperative treatment between 2018 and 2023. Permission to conduct the study was obtained from the Adana City Hospital’s ethics committee.

### 2.2. Determination and Selection of Sample Size

**Figure 1:**
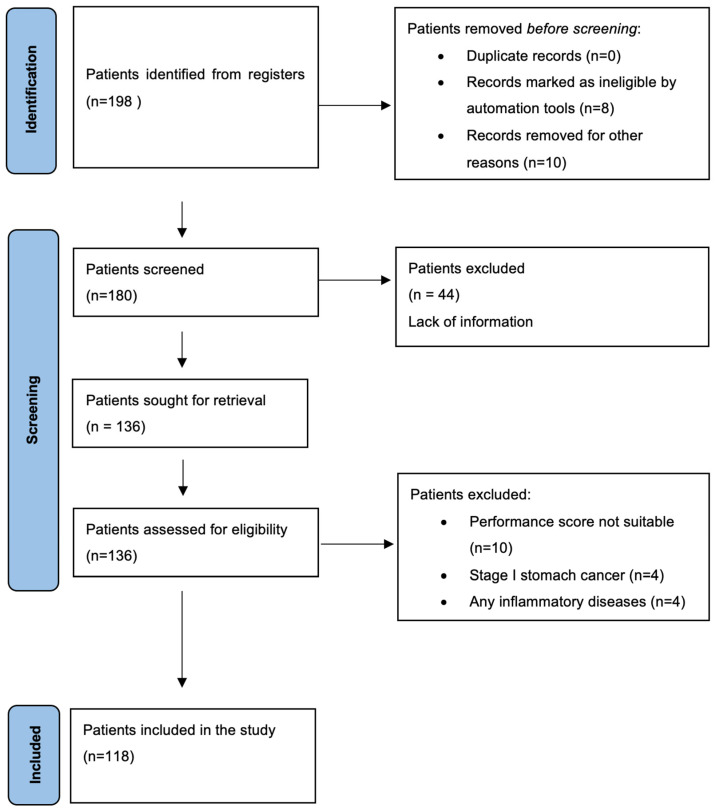
Flow chart of identification of patients via registers.

The sample size analysis with a type 1 error of 0.05, a power of 95%, and an effect size (d) of 0.86 revealed the minimum number to be reached to be 82 patients. As there were no studies in the literature to refer to, the effect size was calculated in a pilot study using our database. A total of 188 early-stage gastric cancer patients applied to 4 centres during the study period; among them, 118 were included in the study based on the inclusion and exclusion criteria (Figure 1).

- Inclusion criteria:
  (I) Patients diagnosed with gastric adenocarcinoma ≥ 18 years of age;
  (II) Patients without metastases;
  (III) Patients with clinical T2 and higher stage treated with perioperative (FLOT) regimen.
- Exclusion criteria:
  (I) Those with organ failure;
  (II) Those whose performance score was not suitable for chemotherapy treatment;
  (III) Those with autoimmune diseases;
  (IV) Those with secondary malignancies;
  (V) Those suffering from inflammatory diseases.

### 2.3. Data Collection

The patient’s age, sex, routine complete blood counts, liver and kidney function tests, albumin value, treatment start date, exitus date, progression date and pathology findings were obtained from the electronic medical registers.

### 2.4. HALP Score

The HALP score was calculated using laboratory test values before the start of perioperative therapy using the following formula:

$$HALP\;score=\frac{{Haemoglobin(g/L)\times Albumin(g/L)\times Lymphocyte\;count(L}^{-1})}{Platelet\;count{(L}^{-1})}$$

### 2.5. Perioperative Treatment Regimen (Every 2 Weeks)

- Oxaliplatin 85 mg/m^2^ in 2 h.
- Docetaxel 50 mg/m^2^ in 1 h.
- Folinic acid 200 mg/m^2^ in 2 h.
- 5-Fluorouracil 2600 mg/m^2^ in 24 h.

### 2.6. Pathological Assessment

Resected gastric tissues were fixed in formaldehyde solution and subsequently embedded in paraffin. Histological tissue sections were obtained and stained with haematoxylin and eosin (H&E). Pathological response status was determined according to the scoring system from the pathology specimen obtained after surgery (Table 1) [16,17].

### 2.7. Statistical Analysis

SPSS 20 (IBM SPSS Statistics for Windows, Version 20.0. Armonk, NY, USA) and JAMOVI 2.3.28 softwares were used for data analysis. Data were presented as numbers, percentages, arithmetic means, standard deviations, and medians. Normality was tested with the Kolmogorov–Smirnov test. The analyses included Student’s *t*-test, Mann–Whitney U test, Pearson’s chi-square test, logistic regression analysis, Kaplan–Meier survival analysis and ROC analysis. Fagan nomogram was used to calculate post-test probabilities (post-test response probability, post-test non-response probability, positive likelihood ratio, and negative likelihood ratio). *p* < 0.05 was considered statistically significant.

## 3. Results

A total of 118 patients with gastric cancer were identified. The mean age of participants was 61.3 ± 11.1, with a male-to-female ratio of 84 (71.2%) to 34 (28.8%). Pathological diagnoses included adenocarcinoma in 95 (80.5%) and signet ring cell carcinoma in 23 (19.5%). The tumour localisations were fundus-cardia in 39 (33.3%), corpus in 38 (32.5%), and antrum-pylorus in 40 (34.2%) patients. Total gastrectomy was performed on 77 (65.3%) patients, and subtotal gastrectomy on 41 (34.7%) patients. Most of the patients were of the intestinal subtype (51.7%), according to the Lauren classification. A pathological response (partial or complete) to perioperative treatment was observed in 81 (68.6%) patients, while no response was seen in 37 (31.4%) patients. The demographic features are summarised in Table 2.

**Figure 2:**
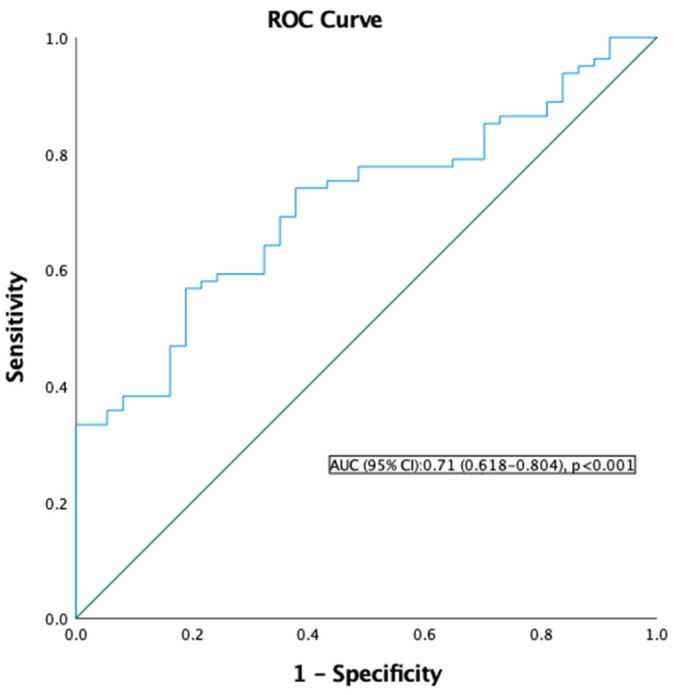
ROC analysis for HALP score. AUC, Area under the ROC curve.

The HALP cutoff was found using the ROC test to predict the pathological response status. After the HALP score cut-off was determined to be 28.9 AUC = 0.71 (*p* < 0.001, Figure 2), the patients were divided into two groups, with low (<28.9) and high (≥28.9) HALP scores.

**Figure 3:**
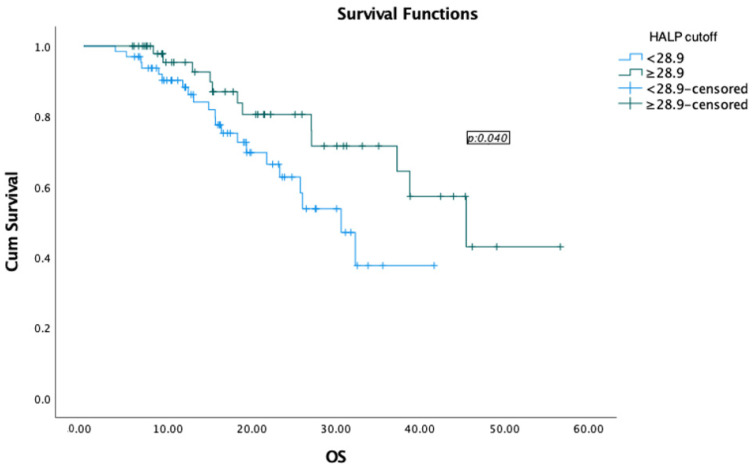
Overall survival according to the HALP score cutoff. OS, Overall survival; cum, cumulative.

Pathological response rates were higher in patients with high HALP scores, and the difference was statistically significant (*p* < 0.001). The progression rate was significantly higher in patients with low HALP scores than in patients with high HALP scores (*p* < 0.001). The median survival time was 45.4 months in the group with high HALP scores and 30.6 months in the group with low HALP scores (Figure 3). The results are summarised in Table 3.

The logistic regression analysis (forward LR method) used to predict the pathological response (non-response versus response) to neoadjuvant treatment gave significant results (omnibus test *p* = 0.002). The dependent variable in the model was the pathological response (predicted = response), while the independent variables were the HALP score, age, sex, tumour localisation, histological subtype, Lauren classification, grade and surgery subtype. The explanatory power of the model was 23.0%. The accuracy of the model was 78.4%. Among the variables included in the model, the HALP score made a significant contribution to the model, with each one-unit increase in this ratio increasing the probability of response 6.5 times (Table 4).

**Figure 4:**
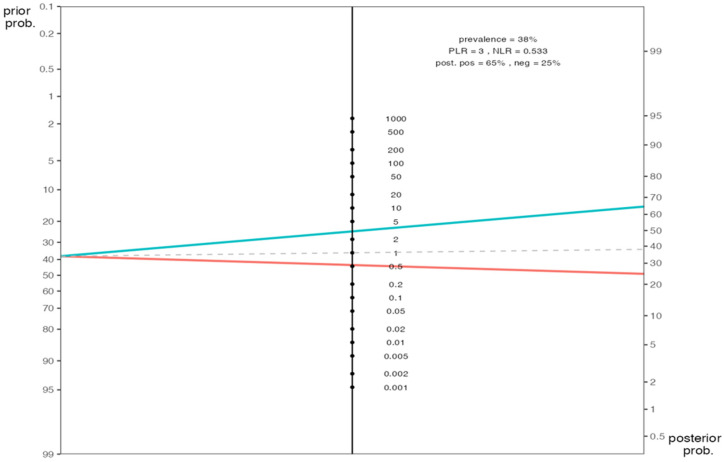
Post-test probability ratios (likelihood ratio) of the HALP score. prob, probability; PLR, positive likelihood ratio; NLR, negative likelihood ratio; post, posterior; pos, positivity; neg, negativity.

Post-test probability ratios (likelihood ratio) of the HALP cutoff value were calculated to predict the pathological response (see Table 1 for the definition of “response” versus “non-response”). Among patients with high HALP scores, the pathological response probability was found to be 64.8%, compared to the pathological non-response rate of 75.4% among low-scored patients. In other words, if the HALP score was 28.9 or above for a patient, our pathological response prediction for a patient increased to 64.8%, while the pathological non-response probability increased to 75.4% (Figure 4).

## 4. Discussion

An immune-nutritional biomarker called HALP has been used to forecast prognosis in cancer patients. The haemoglobin, albumin, lymphocyte, and platelet counts together yield the HALP score, which is a measure of the patient’s nutritional and immune state. Anaemia is a common paraneoplastic condition that is frequently seen in cancer patients, especially in those with gastric cancer, which frequently develops as a result of chronic bleeding linked to stomach cancer [18]. Anaemia is a well-known occurrence that can arise from a number of different pathways in cancer patients. Anaemia of chronic disease (ACD) is a well-researched condition related to cancer [19]. The nutritional status and metabolic needs of a patient influence albumin levels. There is a correlation between low albumin and both inflammation and high nutritional risk [20], rendering the albumin levels a reliable predictor of prognosis for many malignancies [21,22,23]. Therefore, in cachexia-affected cancer patients, elevated serum albumin levels have been linked to lower 1-year death rates [24]. Because lymphocytes are involved in immuno-surveillance, which helps identify and eradicate tumours, it is believed that a decrease in lymphocyte count has a significant impact on prognosis [25]. In the past, lymphocyte scores have been successfully integrated into other prognostic indicators, such as the neutrophil-to-lymphocyte ratio (NLR) and platelet-to-lymphocyte ratio (PLR), to help with the prognosis of different tumours [26]. Moreover, it has been demonstrated that well-described myelosuppression during cytotoxic chemotherapy causes a drop in lymphocyte count. It has been demonstrated that platelets are essential to the ability of cancer to spread [27]. In addition to other inflammatory mediators, platelets also release vascular endothelial growth factor (VEGF) and stimulate tumour angiogenesis [28,29,30,31]. It has also been shown that platelets aid in shielding tumour cells from immune detection. Each of these inflammatory and nutritional markers is taken into account by the HALP score, which provides an overall prognosis for patients with cancer. Haemoglobin, albumin, and lymphocytes are factors that indicate a superior immune–nutritional balance, while platelets are factors that indicate a sick state and are put into the denominator of the HALP calculation. In order to identify individuals with impaired immune–nutritional function and to give a more sophisticated risk stratification, a high HALP score is thought of as a favourable prognostic indication. In our study, patients with high HALP scores had more pathological responses compared to those with low HALP scores. The predictive importance of the HALP score during the preoperative phase in patients with gastric cancer was examined in a study by Wang et al. [32]. They determined a HALP score with a cut-off value of 35.3. Their research showed that when the HALP score was determined before surgery, it was a useful indicator for determining the status of the lymph nodes in patients with gastric cancer. The HALP score, the authors stressed, can be used to tailor the surgical strategy and provide useful data for treatment planning and decision-making. Sargın and Dusunceli conducted a retrospective evaluation of 204 patients diagnosed with gastric cancer. Through the use of ROC analysis, they determined a cut-off of 23.8 for the HALP score. Their study revealed a significant difference in overall survival between patients with high HALP scores and those with low HALP scores; i.e., a high score yielded a better overall survival [33]. In our study, the HALP score cut-off was found to be 28.9 by using ROC analysis, with a significantly longer overall survival in patients with a high HALP score compared to those with a low HALP score.

The main limitation of the study was its retrospective character. The removal of patients from the study due to some missing and incomplete data may also be important in terms of bias.

## 5. Conclusions

According to the study results, the HALP score was found to be a moderately effective tool in predicting pathological response and prognosis following FLOT treatment in patients with locally advanced gastric cancer. The optimum cut-off value for the HALP score in predicting pathological response was determined to be 28.9. When the HALP score was ≥28.9, the estimated probability of achieving a pathological response increased to 64.8%. In the management of patients with locally advanced gastric cancer, this score may serve as a useful biomarker. This simple and cost-effective formula may assist clinicians in estimating pathological responses before the treatment, and in providing patients with prognostic information.

## Figures and Tables

**Table 1:** Pathological response status according to the scoring system from the pathology specimen obtained after surgery.

Score *	Response Status	Interpretation
0	Complete response	Absence of viable cancer cells
1	Near complete response	Presence of individual tumour cells or rare small tumour cell clusters
2	Partial response	Presence of tumour regression with more than individual cells and small tumour cell clusters
3	No response	Absence of tumour regression

* Scores 0, 1 and 2 were classified as the group with a response, while score 3 was classified as the group with no response.

**Table 2:** Demographical features of patients.

Characteristics		Mean ± Standard Deviation or Median [Interquartile Range]
Age		61.3 ± 11.1
Haemoglobin (g/dL)		12.1 ± 1.9
Lymphocyte (L^−1^)		1795.0 [737.0]
Platelet (L^−1^)		277.0 [128.0]
Albumin (g/dL)		3.7 [0.7]
		*n* (%)
Sex	Male	84 (71.2)
	Female	34 (28.8)
Surgery type	Subtotal gastrectomy	41 (34.7)
	Total gastrectomy	77 (65.3)
Histological subtype	Adenocarcinoma	95 (80.5)
	Signet ring cell carcinoma	23 (19.5)
Grade	I	11 (9.4)
	II	60 (51.3)
	III	40 (34.2)
	IV	6 (5.1)
Nodal status	N0	45 (38.1)
	N1	39 (33.1)
	N2	15 (12.7)
	N3	8 (6.8)
	Unknown	11 (9.3)
T-stage	T2	13 (11.0)
	T3	64 (54.2)
	T4	27 (22.9)
	Unknown	14 (11.9)
Lauren classification	Intestinal	61 (51.7)
	Diffuse	37 (31.4)
	Unknown	20 (16.9)
Tumour localisation	Fundus-cardia	39 (33.3)
	Corpus	38 (32.5)
	Antrum-pylorus	40 (34.2)
Response status	Absent	37 (31.4)
	Present	81 (68.6)
Total		118 (100.0)

**Table 3:** The association between HALP score and clinicopathological parameters.

		HALP < 28.9	HALP ≥ 28.9	*p*
Age (median, min–max)		63.0 (37.0–86.0)	61.0 (23.0–78.0)	0.067
		*n* (%)	*n* (%)	
Sex	Male	45 (67.2)	42 (77.8)	0.190
	Female	22 (32.8)	12 (22.2)	
Tumor localisation	Fundus-Cardia	24 (35.8)	17 (32.1)	0.907
	Corpus	21 (31.3)	18 (34.0)	
	Antrum-pylor	22 (32.8)	18 (34.0)	
Surgery type	Subtotal gastrectomy	23 (34.3)	19(35.2)	0.922
	Total gastrectomy	44 (65.7)	35 (64.8)	
Grade	I	5 (7.6)	6(11.1)	0.906
	II	34(51.5)	27(50.0)	
	III	24(36.4)	18(33.3)	
	IV	3(4.5)	3 (5.6)	
Histologic subtype	Adenocarcinoma	56 (83.6)	41 (75.9)	0.294
	Signet ring cell	11 (16.4)	13 (24.1)	
Lauren classification	Intestinal	34 (50.7)	29 (53.7)	0.633
	Diffuse	20 (29.9)	18 (33.3)	
	Unknown	13 (19.4)	7 (13.0)	
Pathological response	Absent	32 (47.8)	7 (13.0)	<0.001 *
	Present	35 (52.2)	47 (87.0)	
Progression status	Absent	35 (52.2)	45(83.3)	<0.001 *
	Present	32 (47.8)	9(16.7)	
Overall survival		30.6 ± 3.1	45.4 ± 7.0	0.040 *

* Indicates statistically significant *p* values.

**Table 4:** Logistic regression analyses for predicting the pathological response.

Predictor	Estimate	*p*	Odds Ratio	Lower	Upper
Intercept	1.98979	0.266	7.314	0.2190	244.307
HALP score (ref: <28.9)					
≥28.9–<28.9	1.87546	<0.001 *	6.524	2.1979	19.364
Age					
Sex (ref:female)	0.00394	0.863	1.004	0.9601	1.050
Male—Female	0.32896	0.546	1.390	0.4781	4.038
Tumor localization (ref:fundus-cardia)					
Corpus—Fundus-cardia	−1.45742	0.022 *	0.233	0.0668	0.811
Antrum-pylor—Fundus-cardia	−1.20124	0.087	0.301	0.0761	1.190
Histological subtype (ref:adenocarcinoma)					
Signet ring cell—Adenocarcinoma	0.65956	0.354	1.934	0.4799	7.793
Lauren_classification: (ref:intestinal)					
Diffuse—Intestinal	−0.61591	0.294	0.540	0.1710	1.706
Grade (ref: Grade I)					
Grade II—Grade I	−0.31267	0.748	0.731	0.1083	4.939
Grade III—Grade I	−1.52241	0.138	0.218	0.0292	1.630
Grade IV—Grade I	0.33604	0.831	1.399	0.0642	30.496
Surgery subtype (ref:subtotal gastrectomy)					
Total gastrectomy—Subtotal gastrectomy	−0.75096	0.228	0.472	0.1391	1.601

* Indicates statistically significant *p* values.

## Data Availability

The data that support the findings of this study are available on request from the corresponding author. The data are not publicly available due to privacy or ethical restrictions.
